# Retraction Note: Identification of novel signaling components in N,N’-Dinitrosopiperazine-mediated metastasis of nasopharyngeal Carcinoma by quantitative phosphoproteomics

**DOI:** 10.1186/s12885-022-09365-y

**Published:** 2022-03-25

**Authors:** Damao Huang, Yuejin Li, Na Liu, Zhenlin Zhang, Zhengke Peng, Chaojun Duan, Xiaowei Tang, Gongjun Tan, Guangrong Yan, Faqing Tang

**Affiliations:** 1 Medical Research Center and Clinical Laboratory, Xiangya Hospital, Central South University, Changsha, 410008 Hunan China; 2 Clinical Laboratory and Medical Research Center, Zhuhai Hospital, Jinan University, Zhuhai, 519000 Guangdong China; 3 Metallurgical Science and Engineering, Central South University, Changsha, 410008 PR China; 4 Institute of Life and Health Engineering, and National Engineering and Research Center for Genetic Medicine, Jinan University, Guangzhou, 510632 China

**Retraction Note: BMC Cancer 14, 243 (2014)**

10.1186/1471-2407-14-243

The Editors have retracted this Article. After publication, concerns were raised regarding the data presented in Figs. 2 and 8. Specifically:

- Fig. 2A(c) appears highly similar to Fig. 2c in [1, retracted] and Fig. 2B(b) in [2].
- Subpanels of Fig. 8A appear highly similar to Fig. 6d and f in [1], Fig. 4a (3rd row, 1st column) in [3], Fig. 8c in [4, retracted] and Fig. 6B(c) in [5].

The authors provided the raw data to address these concerns, but the cell images contained further overlap with the raw data submitted for another paper under investigation. The Editors therefore no longer have confidence in the presented data.

Author Faqing Tang has not specifically stated whether they agree with the retraction. Authors Yuejin Li, Na Liu, Zhenlin Zhang, Zhengke Peng, Chaojun Duan and Gongjun Tan have not responded to any correspondence from the Editor or Publisher about this retraction. The Publisher has not been able to obtain current email addresses for authors Guangrong Yan, Damao Huang and Xiaowei Tang.
